# Protein tau concentration in blood increases after SCUBA diving: an observational study

**DOI:** 10.1007/s00421-022-04892-9

**Published:** 2022-02-10

**Authors:** Anders Rosén, Mikael Gennser, Nicklas Oscarsson, Andreas Kvarnström, Göran Sandström, Helen Seeman-Lodding, Joel Simrén, Henrik Zetterberg

**Affiliations:** 1 Department of Anaesthesia and Intensive Care Medicine, Sahlgrenska University Hospital, Gothenburg, Sweden; 2 Department of Anaesthesia and Intensive Care Medicine, Institute of Clinical Sciences, Sahlgrenska Academy, University of Gothenburg, Gothenburg, Sweden; 3 Swedish Aerospace Physiology Centre, Division of Environmental Physiology, Department of Biomedical Engineering and Health Systems, School of Engineering Sciences in Chemistry, Biotechnology and Health, Royal Institute of Technology, KTH, Stockholm, Sweden; 4 Swedish Armed Forces, Center for Defence Medicine, Gothenburg, Sweden; 5 Clinical Neurochemistry Laboratory, Sahlgrenska University Hospital, Gothenburg, Sweden; 6 Department of Psychiatry and Neurochemistry, Sahlgrenska Academy, University of Gothenburg, Mölndal, Sweden; 7 Department of Neurodegenerative Disease, Institute of Neurology, University College London, London, UK; 8 UK Dementia Research Institute, University College London, London, UK; 9 Hong Kong Center for Neurodegenerative Diseases, Hong Kong, China

**Keywords:** Biomarkers, Brain, Central nervous system, Diving, Diving research, Proteins, Venous gas embolism

## Abstract

**Purpose:**

It is speculated that diving might be harmful to the nervous system. The aim of this study was to determine if established markers of neuronal injury were increased in the blood after diving.

**Methods:**

Thirty-two divers performed two identical dives, 48 h apart, in a water-filled hyperbaric chamber pressurized to an equivalent of 42 m of sea water for 10 min. After one of the two dives, normobaric oxygen was breathed for 30 min, with air breathed after the other. Blood samples were obtained before and at 30–45 and 120 min after diving. Concentrations of glial fibrillary acidic, neurofilament light, and tau proteins were measured using single molecule array technology. Doppler ultrasound was used to detect venous gas emboli.

**Results:**

Tau was significantly increased at 30–45 min after the second dive (*p* < 0.0098) and at 120 min after both dives (*p* < 0.0008/*p* < 0.0041). Comparison of matching samples showed that oxygen breathing after diving did not influence tau results. There was no correlation between tau concentrations and the presence of venous gas emboli. Glial fibrillary acidic protein was decreased 30–45 min after the first dive but at no other point. Neurofilament light concentrations did not change.

**Conclusions:**

Tau seems to be a promising marker of dive-related neuronal stress, which is independent of the presence of venous gas emboli. Future studies could validate these results and determine if there is a quantitative relationship between dive exposure and change in tau blood concentration.

**Supplementary Information:**

The online version contains supplementary material available at 10.1007/s00421-022-04892-9.

## Introduction

It is well-known that diving is not without certain risks. The diver is exposed to increased ambient pressure that causes inert gas accumulation and inhaled gases can, at sufficient depth, exert a noxious effect on the central nervous system (CNS). Oxygen becomes harmful to the CNS when its partial pressure exceeds 160 kPa, equivalent to a depth of about 66 m of seawater (msw) when breathing air. Symptoms of oxygen toxicity range from cognitive and sensory impairment to manifest convulsions (Bitterman 2004). Adverse effects of nitrogen, typically behavioural and intellectual disturbances, become apparent as the partial pressure of nitrogen exceeds 300 kPa (equivalent to a depth of about 28 msw when breathing air) and gradually worsen with increasing pressure (Clarke 2015).

Rapid ascent at the end of a dive may cause barotrauma to the lungs and sinuses. Nitrogen taken up by the body during diving may come out of solution as ambient pressure decreases and form gaseous bubbles in both blood and tissues, which is a common occurrence after diving (Nishi 1981, Eckenhoff 1990). Nitrogen bubbles in the blood can be detected by Doppler ultrasound and are referred to as venous gas emboli (VGE) (Blogg et al. 2014). The amount of VGE in the blood after diving depends on both the amount of accumulated inert gas and decompression stress, but a substantial individual variability exists (Germonpre and Balestra 2017, Papadopoulou et al. 2018). Though VGE is a normal phenomenon after diving, release of dissolved gas as bubbles is considered to be the cause of decompression sickness (DCS) (Eftedal et al. 2007, Blogg et al. 2014). The risk of DCS is correlated to VGE load after diving (Sawatzky 1991, Eftedal et al. 2007). Oxygen breathing employed before (Castagna et al. 2009, Bosco et al. 2010), during (Bosco et al. 2010) and after diving (Blatteau and Pontier 2009) have all been associated with reduced amounts of inert gas bubbles (VGE) in blood, as have active hydration (Gempp et al. 2008), whole body vibration (Germonpre et al. 2010) and sauna-induced heat exposure (Gempp and Blatteau 2010) before diving.

Historically, there have been discussions as to whether repeated, and especially deep diving could be harmful to the nervous system. Several radiological studies involving divers have been published, but the results yielded are hard to interpret, as both the cause and clinical significance of observed CNS lesions often remain uncertain (Knauth et al. 1997, Tetzlaff et al. 1999, Grønning and Aarli 2011, Kohshi et al. 2014, Coco et al. 2019). There have been reports of impaired cognitive performance among both professional and recreational divers without documented DCS, but results from published studies are conflicting. Neuropsychological changes were found in 20% of saturation divers when tested before and after 3.5 years of diving (Vaernes et al. 1989). Saturation divers with self-reported forgetfulness and loss of concentration were found to have mild cognitive deficits when tested objectively and compared to matched controls (Taylor et al. 2006). In a retrospective study, experienced saturation divers did not only have more neurological symptoms but also exhibited more subjective problems with memory and concentration than individuals in a non-diving control group (Todnem et al. 1990, 1991) and experienced recreational divers without a history of DCS have been shown to have a worse short-term memory compared to non-diving control subjects when tested after an average of 12 years diving (Hemelryck et al. 2014). Studies have found inferior neuropsychometric test results among recreational (Balestra et al. 2016), professional (Ergen et al. 2017) and experienced breath-hold divers (Billaut et al. 2018) compared to non-diving controls. Depth and number of dives have been reported to have a negative influence on cognitive performance among recreational divers when tested retrospectively (Slosman et al. 2004). However, contrary to these results, one retrospective study found no difference in neuropsychometric test results when professional divers were compared to matched controls (Cordes et al. 2000), nor was there evidence of neuropsychological impairment among professional, non-saturation divers without a history of DCS when followed-up over 12 years in a longitudinal study (Bast-Pettersen et al. 2015). Post-dive cognitive function studies such as these are hard to interpret, as confounding factors might influence the results, and decreased neuropsychological test performance is not tantamount to neurological impairment.

Increased partial pressure of oxygen (Ferrer et al. 2007, Camporesi Bosco 2014, Bosco et al. 2018) and nitrogen (Bhullar et al. 2016), present during diving, could cause oxidative stress and increased levels of reactive oxygen species (ROS), which could potentially harm cells in the CNS. If diving has a detrimental effect on the brain, it would likely induce changes in the concentration of biochemical markers known to increase in response to CNS trauma or neuronal stress. GFAp is an astrocytic protein involved in several neuronal processes, including synaptic transmission. It has been studied both as a marker of neuronal damage in the context of traumatic brain injury and cerebral haemorrhage when venous concentration increases (Foerch et al. 2012, Zetterberg and Blennow 2016, Gill et al. 2018), and as a marker of degenerative disease (Siracusa et al. 2019). Animal studies suggest that increased immunologic and neuronal activity, as well as neuronal stress could result in changed concentrations of GFAp (Wang and Hatton 2009, Brenner 2014, Femenia et al. 2018). Neurofilament light (NfL) is a neuronal cytoskeletal component found mainly in myelinated subcortical axons. Serum NfL concentrations are increased in patients with cerebral traumas, ranging from sports related concussions (Shahim et al. 2018) to severe traumatic brain injuries (Zetterberg and Blennow 2016, Shahim et al. 2016). Neurodegenerative disorders such as multiple sclerosis and Alzheimer’s disease are associated with increased NfL concentrations in blood (Bergman et al. 2016, Kahlil et al. 2018). Uncomplicated general anaesthesia in conjunction with orthopedic surgery has also been associated with increased blood levels of NfL (Evered et al. 2018).

Tau is a microtubular protein present mainly in unmyelinated cortical axons but also, to a lesser extent, in the liver, kidneys and testes. Tau could be both passively released as a result of manifest axonal damage (Zetterberg and Blennow 2016) and actively secreted in connection to increased neuronal activity in response to stress (Sato et al. 2018). Neurodegenerative diseases, sports related concussions, boxing and traumatic brain injuries are all associated with increased tau levels in blood (Zetterberg et al. 2006, Neselius et al. 2012, Zetterberg and Blennow 2016, Mattson 2017, Shahim et al. 2018). As with NfL, increased blood concentrations of tau have been found after uneventful general anaesthesia in conjunction with orthopedic surgery (Evered et al. 2018). Physiological stressors such as protracted apnea among breath-hold divers (Gren et al. 2016) and high intensity interval training (Battista et al. 2018) have both been associated with increased tau levels in blood. A small pilot study found increased serum tau levels in blood after repeated deep, open sea diving (Rosén et al. 2019). In contrast, tau levels were not increased in cerebrospinal fluid in a small study on divers with DCS, though only one of seven patients in the study had CNS symptoms (Shahim et al. 2015). In addition, serum tau did not increase in response to nitrox saturation exposure in a study on submariners (Rosén et al. 2020).

The present study tested the hypothesis that diving to 42 msw would, via neuronal stress, incur a change in GFAp, NfL and tau. It was also investigated whether breathing normobaric oxygen after diving changed the amount of GFAp, NfL and tau in blood.

## Materials and methods

The study was conducted at the Swedish Armed Forces (SwAF) naval base in Karlskrona, Sweden, during June 2015 and November 2018, as part of a project where subjects performed identical dives breathing either air or normobaric oxygen after decompression. The study was prospective, observational, registered at ClinicalTrials.gov (NCT02468752) and approved by Swedish ethical review authorities. Participants were recruited among professional divers from the SwAF, the Swedish Coast Guard and the Swedish Police. Written consent was obtained from all subjects before the start of the study.

Altogether, 33 professional divers were recruited. Data concerning age, gender, weight, height, medication and prior DCS were collected from all subjects. A water-filled hyperbaric chamber was used to simulate diving. After entering the chamber, the study subjects immersed themselves in water and rested in a horizontal position close to the bottom of the chamber for the duration of the experiment. All study subjects breathed air using an open circuit system.

The chamber was electronically pressurized to 521 kPa (equivalent of 42 msw) for 10 min by an external operator. Compression speed was 200 kPa/min, and decompression speed 90 kPa/min (equivalent to 9 msw/min), with a safety stop made at 151 kPa (equivalent to 5 msw) for 3 min. After surfacing, either oxygen or air was breathed for 30 min using tight-fitting face masks with demand valves. The breathing gas a particular study subject used after the first dive was randomly chosen by an independent operator and hence unknown to both study subjects and study personnel. In June 2015 (16 subjects, 32 dives), oxygen or air breathing commenced immediately after surfacing, while in November 2018 (16 subjects, 32 dives), mask breathing was deliberately delayed by 15 min. After a predetermined interval of 48 h, all subjects performed an identical second dive, with a switch of the breathing gases afterwards. Study subjects and all study personnel were unaware of the breathing gas used after a particular dive throughout the trial.

Immediately after surfacing, the subjects were monitored for VGE by a blinded operator using precordial Doppler ultrasound (DBM9008, Techno Scientific Inc, Ontario, Canada). Measurements were made every five minutes during the first 30 min and every 15 min thereafter for a further 90 min. Venous gas emboli were measured using the Kisman Masurel (KM) grading system, which is an ordinal scale based on categorical data describing amplitude, frequency and duration of VGE (Kisman 1978a). Maximal KM grades were recorded for every subject after each dive (VGE_max_). For each subject, the Kisman Integrated Severity Score (KISS) algorithm (Kisman et al. 1978b, Jankowski et al. 2004) was used to convert all KM grades collected from 0–30 min and 0–120 min after diving into individual integrated scores (KISS_30min_ and KISS_120min_).

Venous blood samples were collected 1–3 h before the first dive (sample 1, baseline), after mask breathing had ended (sample 2, 30–45 min after diving) and 120 min after diving (sample 3). For the second dive, the same blood protocol was performed, with the resulting samples named 4–6. Besides providing a baseline value before the second dive, Sample 4 was also used as the last (48 h after diving) in the series of samples taken after the first dive.

Plasma EDTA tubes (Vacuette nr 454410, Hettish Labinstrument AB, Sweden) were used for blood collection and samples were centrifuged for 15 min at 2500 rpm and 4° centigrade (Sorvall ST 8/8R centrifuge, Thermo Scientific, Germany). Directly after centrifugation, aliquots of 500 μL plasma were frozen at −18° centigrade for 1–4 days, transported on dry ice and then stored at −78° centigrade until analysed.

GFAp, NfL and tau concentrations were measured using the Human Neurology 4-Plex A assay on an HD-1 Single molecule array (Simoa) instrument (Quanterix, Lexington, MA, USA). The 4-Plex assay also included ubiquitin carboxy-terminal hydrolase L-1 (UCH-L1), which was not further assessed in this study. All samples were analysed together in one batch. For quality control (QC) samples with GFAp concentrations of 87.2 pg mL^−1^ and 465.2 pg mL^−1^, coefficients of variation (CVs) were 7.3% and 3.2% respectively, for QC samples with NfL concentrations of 8.7 pg mL^−1^ and 46.2 pg mL^−1^ CVs were 9.2% and 3.7% and for quality control (QC) samples with tau concentrations of 1.0 pg mL^−1^ and 2.6 pg mL^−1^ CVs were 4.3% and 7.9%, respectively.

### Statistics

Compilation of demographic data was performed using IBM SPSS® v24 (IBM, Armonk, NY, USA). Statistical analyses regarding absolute changes in GFAp, NfL and tau were performed by an independent statistical company using SAS® v9.4 (Cary, NC, USA). Concentrations of GFAp, NfL and tau (pg·mL^−1^) were presented with both mean and median values with standard deviation (SD) and range (minimum and maximum values) stated. Fisher’s non-parametric permutation test for paired observations was used in analyses within groups and Fisher’s non-parametric permutation test in analyses between groups. Mean difference with 95% confidence interval (CI) was considered the main result. All significance tests were two-sided with a significance level of 5%. Spearman’s rank correlation test was used to assess if there were any correlations between GFAp, NfL or tau concentrations and the presence of VGE. A positive or negative Spearman correlation coefficient greater than 0.8 was used as a limit to accept correlation between variables. KISS values and relative changes in GFAp, NfL and tau (%) were computed using Microsoft© Office Excel 2018 (Microsoft Corporation, Redmond WA, USA).

### Missing data

One subject from the June 2015 cohort was excluded from the study due to dysbarism at the start of his first dive. His demographic data and Sample 1 results were omitted from all compilations and analyses. All remaining 32 divers completed two dives. There were no missing data for GFAp, NfL and tau. All VGE data were collected according to the protocol.

## Results

### Demographics

Among the 32 subjects that completed the study, 31 were males and one was female. Mean age was 37.9 years [standard deviation (SD) 8.1, range 26–55 years) and mean body mass index (BMI) was 25.4 (SD 1.7, range 21.4–29.2). Two subjects had experienced shoulder pain, possibly a sign of decompression sickness (DCS), earlier in their diving career but none had been treated in a hyperbaric oxygen chamber. One subject was prescribed antihypertensive medication with losartan, another for unknown reasons used diclofenac once between the two dives.

### Biomarkers of neuronal injury

#### Effect of breathing oxygen after diving

To assess if breathing oxygen after diving had an effect on GFAp, NfL or tau concentrations, samples obtained after breathing oxygen (samples 2 and 3 or 5 and 6) were compared to matching samples after breathing air (samples 5 and 6 or 2 and 3, respectively). A potential period effect was adjusted for by using Fisher’s non-parametric test to analyse changes in GFAp, NfL and tau concentrations between the first and second dive, comparing subjects who breathed air to subjects who breathed oxygen after the first dive. Oxygen breathing did not influence obtained NfL or tau values. No significant differences were found for these proteins at any point. At 30–45 min after diving there were no differences in GFAp results between divers breathing oxygen or air, but results for GFAp were higher 120 min after diving when breathing air compared to oxygen. Results for analyses of the effect of oxygen breathing on GFAp, NfL and tau are presented in Table 1.

**Table 1:** Differences in GFAp, NfL and tau values after breathing oxygen compared to air-adjusted for period effect

Absolute change from sample 2 to sample 5 at 30–45 min after first or second dive					
	Breathing oxygen after first dive (*n* = 18)	Breathing air after first dive (*n* = 14)	*p*-value	Arithmetic mean difference between groups	Effect of breathing oxygen adjusted for period effect − mean difference between groups
GFAp (pg mL^−1^)	1.06 (9.15)	−2.47 (11.98)	0.35	3.53 (−4.15; 11.07)	1.76 (−2.08; 5.54)
	0.39 (−18.4; 14.05)	−0.35 (−25.38; 19.47)			
	(−3.46; 5.57)	(−9.42; 4.45)			
NfL (pg mL^−1^)	−0.035 (1.061)	0.249 (2.159)	0.65	−0.284 (−1.429; 0.851)	−0.14 (−0.72; 0.43)
	0.01 (−3.348; 1.424)	−0.233 (−2.477; 6.625)			
	(−0.563; 0.446)	(−0.816; 1.503)			
Tau (pg mL^−1^)	0.180 (0.920)	0.275 (0.728)	0.75	−0.095 (−0.695; 0.529)	−0.05 (−0.35; 0.26)
	−0.163 (−1.034; 2.315)	0.147 (−0.657; 2.236)			
	(−0.273; 0.640)	(−0.114; 0.700)			

Absolute change from sample 3 to sample 6 at 120 min after first or second dive					
	Breathing oxygen after first dive (*n* = 18)	Breathing air after first dive (*n* = 14)	*p*-value	Arithmetic mean difference between groups	Effect of breathing oxygen adjusted for period effect − mean difference between groups
GFAp (pg mL^−1^)	4.92 (8.80)	−2.70 (11.22)	0.039	7.62 (0.35; 14.78)	3.81 (0.18; 7.39)
	3.93 (−9.31; 22.32)	1.01 (−23.39; 17.42)			
	(0.58; 9.33)	(−9.21; 3.75)			
NfL (pg mL^−1^)	−0.029 (0.780)	−0.203 (0.796)	0.54	0.175 (−0.394; 0.749)	0.09 (−0.20; 0.38)
	−0.189 (−1.54; 1.985)	−0.081 (−2.339; 0.621)			
	(−0.404; 0.357)	(−0.664; 0.219)			
Tau (pg mL^−1^)	−0.138 (1.509)	0.285 (1.318)	0.43	−0.423 (−1.459; 0.603)	−0.21 (−0.73; 0.30)
	−0.091 (−4.539; 3.702)	0.233 (−1.792; 4.229)			
	(−0.855; 0.571)	(−0.363; 1.047)			

Mean (standard deviation)/median (range)/(95% CI for mean using the inversion of Fisher’s non-parametric permutation test) are presented. For comparison between groups the Fisher’s non-parametric permutation test was used. Mean difference between groups is presented with a 95% confidence interval. The arithmetic mean difference between groups equals twice the effect of given treatment (oxygen or air)

### Effect of prior diving (48 h before sample 4)

The difference between samples obtained before each dive (samples 1 and 4) was compared separately for subjects breathing oxygen and air after their first dive, to see if a residual effect was present. No significant differences in GFAp, NfL and tau concentrations immediately before the two dives were found, suggesting that neither diving nor oxygen breathing 48 h prior to the second dive had an effect on the results obtained. The results are presented in Table 2.

**Table 2:** Differences in GFAp, NfL and tau values before each dive—estimation of carryover effect

Absolute difference between samples 1 and 4						
	Subjects breathing oxygen after first dive (*n* = 18)	*p*-value within group	Subjects breathing air after first dive (*n* = 14)	*p*-value within group	Mean absolute difference between groups	*p*-value between groups
GFAp (pg mL^−1^_)_	−0.04 (12.77)	0.99	−6.88 (15.38)	0.12	6.83 (−3.14; 17.12)	0.18
	−4.52 (−20.05; 27.82)		−8.36 (−33.76; 15.47)			
	(−6.29; 6.39)		(−15.90; 2.06)			
NfL (pg mL^−1^_)_	0.02 (1.16)	_0.95_	0.60 (2.81)	0.62	−0.58 (−1.97; 0.80)	0.52
	−0.28 (−1.56; 3.47)		−0.22 (−1.33; 9.62)			
	(−0.520; 0.599)		(−0.65; 2.21)			
Tau (pg mL^−1^_)_	−0.04 (0.52)	0.75	0.01 (0.48)	0.96	−0.05 (−0.42; 0.31)	0.78
	−0.08 (−1.36; 0.86)		0.16 (−0.96; 0.56)			
	(−0.30; 0.21)		(−0.27; 0.29)			

Mean (standard deviation)/median (range)/(95% CI for mean using the inversion of Fisher’s non-parametric permutation test) is presented. For comparison within groups Fisher’s non-parametric permutation test for matched pairs was used. The confidence interval for the mean difference between groups was based on Fishers non-parametric permutation test

### Effect of diving

GFAp, NfL and tau blood levels after diving were analysed linearly, comparing changes between sample 1 and samples 2–4 as well as between samples 4 and samples 5–6 in sequence (Table 3), and also according to breathing gas used, ignoring order of the dives (Table 4).

**Table 3:** Changes in GFAp, NfL and tau after diving

Sample 1: before first dive	Absolute value (*n* = 32)	Absolute change compared to sample 1 (*n* = 32)	*p*-value
GFAp (pg mL^−1^)	60.3 (26.2)		
	51.9 (31.2; 139.2)		
NfL (pg mL^−1^)	7.84 (7.72)		
	6.12 (3.4; 47.79)		
Tau (pg mL^−1^)	1.70 (0.89)		
	1.5 (0.4; 4.3)		

Sample 2: 30–45 min after first dive	Absolute value (*n* = 32)	Absolute change compared to sample 1 (*n* = 32)	*p*-value
GFAp (pg mL^−1^)	56.6 (24.0)	−3.69 (10.08)	0.045
	49.4 (29; 139)	−2.90 (−30.21; 20.22)	
		(−7.29; −0.06)	
NfL (pg mL^−1^)	7.75 (8.28)	−0.09 (0.92)	0.61
	5.66 (3.21; 51.01)	−0.24 (−1.29; 3.22)	
		(−0.40; 0.24)	
Tau (pg mL^−1^)	1.89 (0.84)	0.18 (0.73)	0.17
	1.86 (0.39; 3.68)	0.20 (−2.08; 2.22)	
		(−0.08; 0.44)	

Sample 3: 120 min after first dive	Absolute value (*n* = 32)	Absolute change compared to sample 1 (*n* = 32)	*p*-value
GFAp (pg mL^−1^)	56.5 (20.9)	−3.82 (12.88)	0.11
	55.4 (26.5; 128.5	−3.31 (−40.28; 15.25)	
		(−8.52; 0.87)	
NfL (pg mL^−1^)	8.11 (8.33)	0.271 (1.11)	0.18
	6.4 (3.05; 51.61)	0.11 (−1.68; 3.83)	
		(−0.13; 0.67)	
Tau (pg mL^−1^)	2.18 (1.47)	0.48 (1.03)	0.0008
	1.84 (0.45; 8.13)	0.27 (−1.03; 5.21)	
		(0.15; 0.82)	

Sample 4: before second dive (48 h after the first dive)	Absolute value (*n* = 32)	Absolute change compared to sample 1 (*n* = 32)	*p*-value
GFAp (pg mL^−1^)	57.3 (20.6)	−3.03 (14.16)	0.24
	58.3 (33; 135.7)	−5.83 (−33.76; 27.82)	
		(−8.18; 2.14)	
NfL (pg mL^−1^)	8.11 (8.43)	0.27 (2.03)	0.55
	6.02 (3.6; 51.25)	−0.28 (−1.56; 9.62)	
		(−0.38; 0.97)	
Tau (pg mL^−1^)	1.68 (0.81)	−0.02 (0.50)	0.81
	1.54 (0.58; 3.16)	0.01 (−1.36; 0.86)	
		(−0.20; 0.16)	

Sample 5: 30–45 min after second dive	Absolute value (*n* = 32)	Absolute change compared to sample 4 (*n* = 32)	*p*-value
GFAp (pg mL^−1^)	56.2 (19.5)	−1.14 (8.34)	0.45
	55 (31.5; 127.6)	−0.88 (−14.61; 13.69)	
		(−4.21; 1.91)	
NfL (pg mL^−1^)	7.84 (7.64)	−0.27 (2.07)	0.48
	6.16 (3.44; 47.66)	−0.11 (−7.54; 6.38)	
		(−1.01; 0.45)	
Tau (pg mL^−1^)	2.11 (1.32)	0.42 (0.92)	0.0098
	1.89 (0.53; 5.27)	0.24 (−1.67; 3.09)	
		(0.10; 0.76)	

Sample 6: 120 min after second dive	Absolute value (*n* = 32)	Absolute change compared to sample 4 (*n* = 32)	*p*-value
GFAp (pg mL^−1^)	58.1 (20.3)	0.80 (10.06)	0.65
	57.3 (30.5; 138)	0.95 (−18.24; 22.63)	
		(−2.81; 4.39)	
NfL (pg mL^−1^)	8.01 (8.38)	−0.10 (1.49)	0.84
	6.12 (3.05; 51.95)	0.19 (−7.31; 1.94)	
		(−0.61; 0.34)	
Tau (pg mL^−1^)	2.23 (1.56)	0.54 (1.20)	0.0041
	1.96 (0.6; 6.74)	0.12 (−0.90; 4.91)	
		(0.14; 0.97)	

Mean (SD)/median (min; max)/(95% CI for mean using the inversion of Fisher’s non-parametric permutation test) is presented. For comparison within groups the Fisher’s non-parametric permutation test for matched pairs was used

**Table 4:** Changes in GFAp, NfL and tau—breathing air or oxygen after diving

Breathing oxygen after diving		
30–45 min after diving	Absolute change (*n* = 32)	*p*-value
GFAP (pg mL^−1^)	−1.76 (9.72)	0.31
	−1.26 (−22.73; 13.69)	
	(−5.24; 1.75)	
NfL (pg mL^−1^)	−0.24 (2.014)	0.52
	−0.28 (−7.54; 6.38)	
	(−0.95; 0.452)	
Tau (pg mL^−1^)	0.30 (0.77)	0.03
	0.22 (−2.08; 2.22)	
	(0.02; 0.57)	

120 min after diving	Absolute change (*n* = 32)	*p*-value
GFAP (pg mL^−1^)	−1.99 (10.65)	0.31
	−2.43 (−34.37; 15.25)	
	(−5.81; 1.83)	
NfL (pg mL^−1^)	−0.08 (1.637)	0.83
	0.08 (−7.31; 3.83)	
	(−0.64; 0.45)	
Tau (pg mL^−1^)	0.60 (1.29)	0.0014
	0.24 (−1.03; 5.21)	
	(0.16; 1.05)	

Breathing air after diving 30–45 min after diving	Absolute change (*n* = 32)	*p*-value
GFAP (pg mL^−1^)	−3.07 (8.89)	0.06
	−2.41 (−30.21; 20.22)	
	(−6.27; 0.11)	
NfL (pg·mL^−1^)	−0.12 (1.03)	0.53
	−0.01 (−3.59; 1.83)	
	(−0.49; 0.25)	
Tau (pg·mL^−1^)	0.307 (0.903)	0.06
	0.20 (−1.67; 3.09)	
	(−0.01; 0.63)	

120 min after diving	Absolute change (n = 32)	*p*-value
GFAP (pg mL^−1^)	−1.02 (12.82)	0.66
	0.04 (−40.28; 22.63)	
	(−5.68; 3.59)	
NfL (pg mL^−1^)	0.24 (0.89)	0.12
	0.27 (−1.27; 1.94)	
	(−0.07; 0.56)	
Tau (pg mL^−1^)	0.42 (0.90)	0.0034
	0.21 (−0.90; 4.18)	
	(0.12; 0.74)	

For continuous variables mean (SD)/median (min; max)/(95% CI for mean using the inversion of Fisher’s non-parametric permutation test) are presented. For comparison within groups the Fisher’s non-parametric permutation test for matched pairs was used

### GFAp

When divers were analysed together irrespective of post-dive breathing gas, GFAp concentrations were significantly decreased at 30–45 min after the first dive but not at 120 min post-dive. No significant changes in GFAp concentrations were observed after the second dive (Table 3). In addition, there were no significant changes in GFAp after either dive when analysed based on post-dive breathing gas (Table 4).

### NfL

Variations in mean NfL concentrations observed after the two dives did not reach statistical significance (Tables 3 and 4).

### Tau

Mean protein tau concentrations increased after each dive. When all divers were analysed together, irrespective of breathing gas used after a certain dive, the changes were statistically significant 120 min after both dives, and at 30–45 min after the second dive. Tau concentrations decreased between the dives with mean tau concentrations obtained prior to both dives being almost identical (Table 3). When changes in tau were analysed based on breathing gas used, mean levels were significantly increased at 30–45 min following oxygen breathing and at 120 min regardless of breathing gas used (Table 4).

Mean tau had increased by 29.1% (SD 44.7%) and 33.9% (SD 81.7%) at 120 min after the first and second dive, respectively. When all 64 dives were analysed together, the mean tau increase at 120 min after diving was 31.5% (SD 66.4%). In one diver, tau increased by 428% after the second dive. If this outlier value was omitted, the tau increase after the second dive was 21.2% (SD 41.5%), and was 25.2% (SD 43.7%) at 120 min for the remaining 63 dives taken together.

### Correlation between markers of neuronal injury and venous gas emboli

**Fig. 1:**
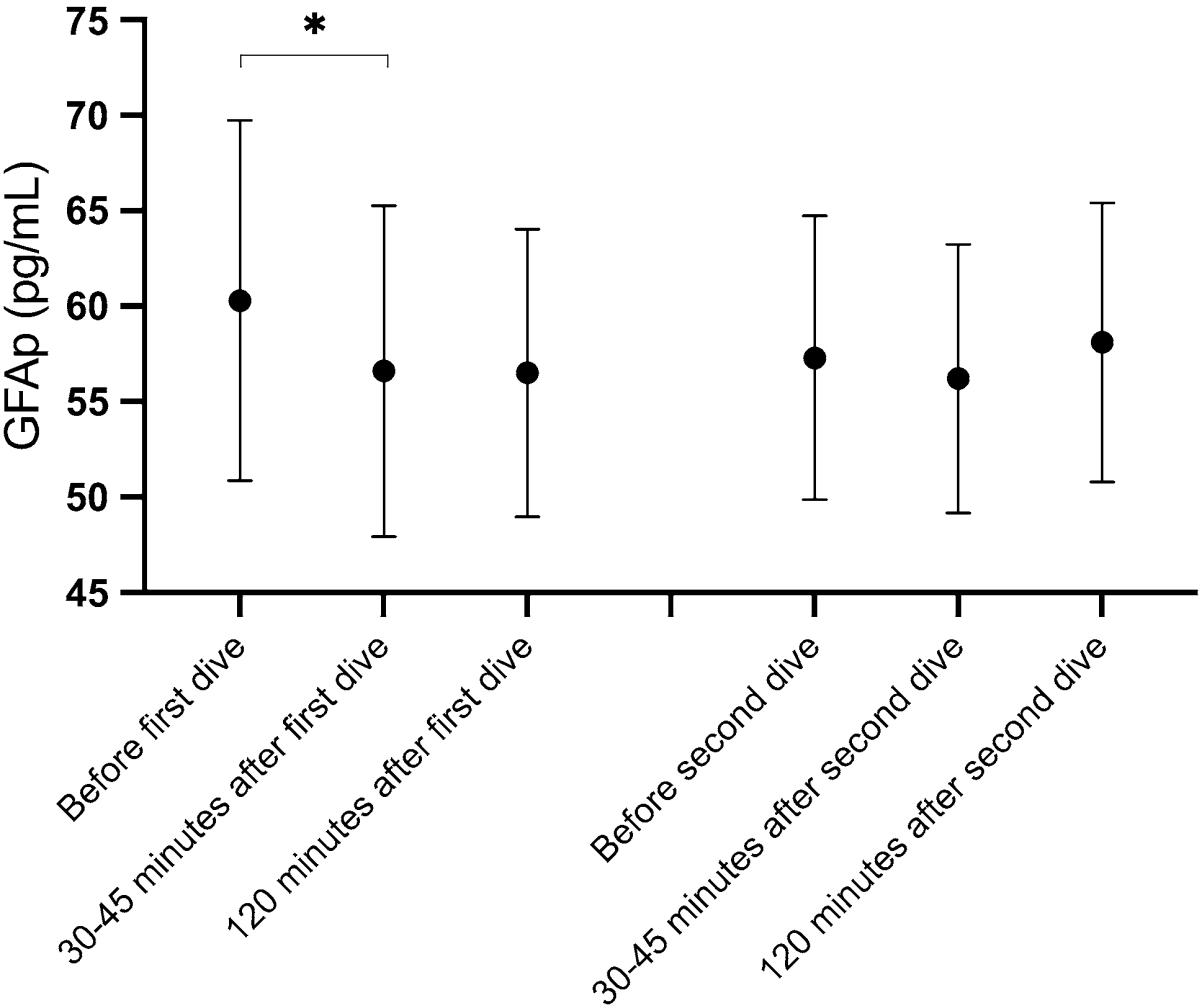
GFAp concentrations after diving. Mean GFAp concentrations with 95% confidence intervals

**Fig. 2:**
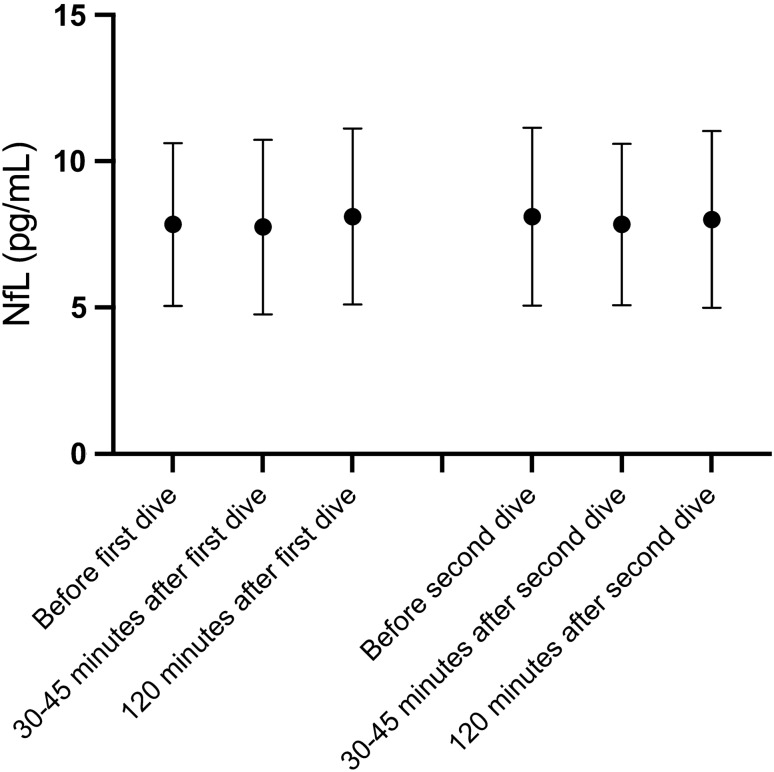
NfL concentrations after diving. Mean NfL concentrations with 95% confidence intervals

**Fig. 3:**
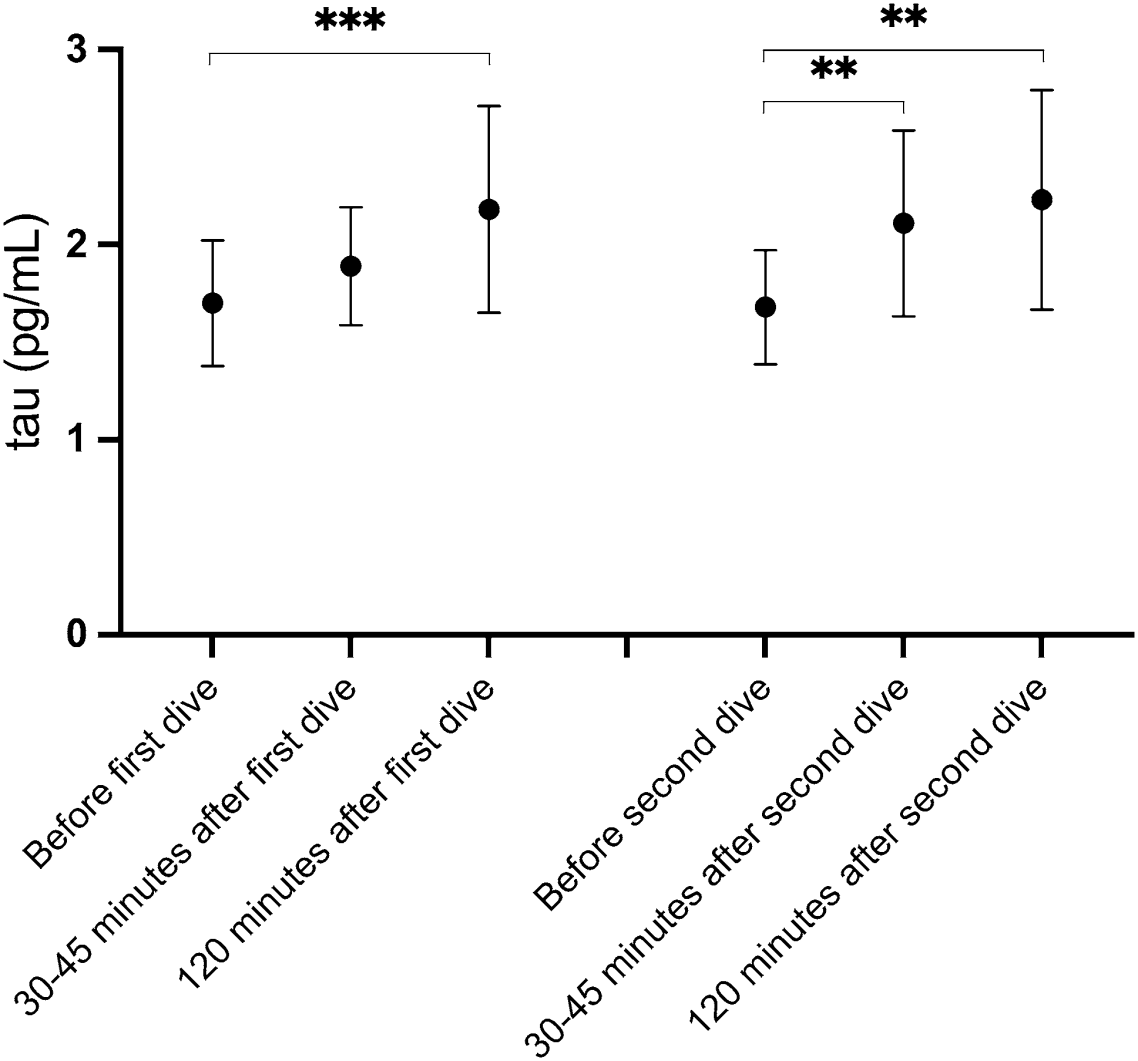
Protein tau concentrations after diving. Mean tau concentrations with 95% confidence intervals

Absolute values for GFAp, NfL and tau concentrations at 30–45 and 120 min after each dive as well as changes in GFAp, NfL and tau concentrations at these points were tested for correlation with matching VGE_max_, and KISS_30min_ or KISS_120min_ values, respectively. No correlations were found. Results are presented in Appendices 1 and 2 (Figs. 1, 2, 3).

## Discussion/conclusions

The present study found that tau levels in blood increased after a dive made to an equivalent of 42 msw depth. When an identical dive was performed 2 days later, the increases in tau observed after the first dive were reproduced. Tau changes seemed to be fast, with a measurable increase as soon as 30–45 min after diving, with yet higher tau levels found after 120 min. The design of the present study did not allow us to determine peak tau levels, or determine when they appeared, but after 48 h tau blood levels had returned to values obtained before the first dive.

Breathing normobaric oxygen after diving did not affect the tau blood levels obtained. Hence, tau changes could be analysed without regard to the breathing gas used after each dive. Nevertheless, the results were strengthened by the fact that increases in tau concentrations seen 120 min after diving also remained statistically significant for both groups when dives with oxygen and air breathing afterwards were analysed separately. There was no correlation between absolute tau concentrations or their changes and VGE loads, which indicates that tau release is not affected by the presence of VGE.

Tau blood levels rise after brain damage and neuronal cell death, with median concentrations of 49.5 pg mL^−1^ observed at 48 h after cardiac arrest among patients with poor neurological outcome (Mattson 2017), whereas in the present study, tau concentrations were 2.2 pg mL^−1^ 120 min after diving. The physiological mechanism that causes tau levels to increase, albeit to a lesser degree, after presumed neuronal stress without manifest injury to the CNS has not been identified. Breathing normobaric oxygen after diving did not affect tau blood levels in the present study, but it is still possible that exposure to supranormal partial pressures of oxygen might affect tau levels. Other possible causes of increased tau blood levels are increased ambient pressure per se, changes in cerebral perfusion during immersion, or compression stress. As there was no correlation between VGE and tau levels, decompression stress seems unlikely to have influenced the results.

Trimix breathing gas is used for deep dives and contains oxygen, helium and nitrogen. Tau increased by 98.8% in a small pilot study where ten divers performed repeated deep dives between 52–90 msw over 4 days using trimix (Rosén et al. 2019), with an oxygen partial pressure of 130 kPa during the dive and up to 160 kPa during decompression; nitrogen pressures at depth were around 176–193 kPa. In the present study the partial pressures of oxygen and nitrogen at depth were 109 kPa and 406 kPa, respectively. The trimix study found no correlation between VGE loads and tau concentrations, either in terms of their absolute values or their changes. The larger relative increase in tau blood levels after diving in the trimix study compared to the present study could be due to differences in dive depths, oxygen partial pressures, breathing gas, and number of dives between the two studies.

In another study, when submariners were exposed to a pressure equivalent to 30 msw (401 kPa) for 36 h in a dry hyperbaric chamber, then followed by slow decompression over a further 70 h, no significant change in tau blood concentration was seen (Rosén et al. 2020). The submariners experienced a lower ambient pressure than the divers in the present study, but their duration of exposure was longer. Tau was sampled before exposure, at 33–34 h of exposure, and after exposure had ended. No samples were obtained at either 30–45 or 120 min of exposure, making comparison between the present study and the submariner study difficult; it is possible that tau levels may have increased after initial pressurization to 30 msw (401 kPa) and then decreased, reaching baseline levels before a sample was obtained at 33–34 h. In the submariner study, the maximum oxygen partial pressure was 50 kPa, with a maximum nitrogen partial pressure of about 350 kPa, and the rate of decompression was 0.375–0.5 msw/h; this rate is much slower than that of the present study (9 msw/min).

It could be speculated that the changes in tau seen after diving were caused by oxidative stress and increased levels of ROS but the fact that tau increases were unaffected by normobaric oxygen breathing during 30 min after diving, an exposure three times longer and just slightly less hyperoxic than the dive itself, makes such a mechanism less plausible. Higher nitrogen partial pressures in this study did not coincide with larger tau increases, compared to the trimix study.

Different preconditioning techniques have been shown to decrease VGE load after diving though none of them were employed in this study, which makes it impossible to judge their potential effect on tau concentrations. Oxygen breathing before diving would probably have reduced VGE load but at the same time caused oxidative stress to the diver. As there was no association between VGE and tau, and the effect of oxidative stress on tau is uncertain, it is questionable if oxygen breathing before diving would have affected the results. Possible effects of other preconditioning techniques such as sauna-induced heat exposure or whole-body vibration remain to be elucidated.

A difference in GFAp blood concentrations 120 min after diving was observed when samples from divers breathing oxygen were compared to their paired air samples, which suggests that oxygen might have influenced the change in GFAp blood levels obtained at this point. Therefore, it was not prudent to analyse changes in GFAp blood levels without regard to breathing gas used after the dive. However, when GFAp samples were analysed in two separate groups based on breathing gas, GFAp blood levels were not significantly changed at any point, though a decrease seen in GFAp 30–45 min after dives followed by air breathing almost reached significance (*p* = 0.06). When all dives were analysed irrespective of breathing gas post-dive, GFAp was significantly decreased at 30–45 min after the first dive but not at the same point after the second. There were no significant changes at 120 min after either dive. These disparate results regarding changes in GFAp blood levels after diving suggest that it would not be useful as a marker of dive-related, presumably neuronal, stress. There was no correlation between GFAp concentrations, or their changes and VGE loads.

NfL levels in blood did not change significantly in the present study, which is consistent with the aforementioned studies on deep repeated diving and prolonged hyperbaric exposure of submariners. The results do not support neuroaxonal injury, although the slow dynamics of NfL, with maximum increases seen later than seven days after an insult (Shahim et al. 2016), makes it a less well-suited marker in the setting of this study. NfL levels in blood were not influenced by oxygen breathing after diving or correlated to VGE load.

Dehydration is common after diving and could cause increased concentrations of proteins measured in blood but this parameter was not assessed, which is a shortcoming of this study. However, as tau increased while NfL remained unchanged and GFAp either remained unchanged or decreased, significant dehydration seems unlikely. Active hydration before diving is a preconditioning technique (Gempp et al. 2008) which theoretically may cause a decrease in venous protein concentrations after diving, but it was not employed in the present study.

Samples were taken only up to 120 min after each dive. Continued sampling may have yielded more detailed data on changes in tau concentration after diving, potentially making it possible to determine maximum tau values in blood after each dive.

In this study, tau blood levels increased after diving. The use of repeated, uniform dive exposures is a strength of the study and the fact that tau increases were similar after the first and the second dives makes the results convincing. Tau concentrations in blood were not correlated to VGE. Based on these results, as well as the aforementioned pilot study on deep trimix diving, tau seems to be a promising marker of dive-related, presumably neuronal, stress.

A larger study where subjects perform repeated dives to different depths and durations is necessary both to validate these results and to establish if there is a quantitative relationship between dive exposure and tau levels in blood. Blood sampling should ideally be frequent and continued for at least hours after each dive.

## Supplementary Information

Below is the link to the electronic supplementary material.

Appendix 1: Spearmans correlation for GFAP, NfL, tau and their changes from baseline with VGE _max_ and KISS_30min_ and KISS_120min_—breathing oxygen or air after diving (DOCX 17 KB)

Appendix 2: Spearmans correlation for GFAP, NfL, tau and their changes with VGE _max_ and KISS_30min_ or KISS_120min_ after each dive (DOCX 17 KB)

## Abbreviations

BMI: Body mass index
CNS: Central nervous system
CV: Coefficient of variation
DCS: Decompression sickness
GFAp: Glial fibrillary acidic protein
KISS: Kisman integrated severity score
KM: Kisman Masurel
kPa: Kilopascal
msw: Meters of seawater
NfL: Neurofilament light
Simoa: Single molecule array
SwAF: Swedish armed forces
tau: Protein tau
UCH-L1: Ubiquitin carboxy-terminal hydrolase L-1
VGE: Venous gas emboli
QC: Quality control

## Declarations

### Availability of data and material

The datasets generated and analysed during the current study are available from the corresponding author on reasonable request.
