# Supplementary material for: Protein tau concentration in blood increases after SCUBA diving: an observational study
Source: Eur J Appl Physiol. 2022 Feb 10;122(4):993–1005. doi: 10.1007/s00421-022-04892-9 (PMC8926952; doi:10.1007/s00421-022-04892-9)
Supplement: Supplementary file 1 — Appendix 1: Spearmans correlation for GFAP, NfL, tau and their changes from baseline with VGE max and KISS30min and KISS120min—breathing oxygen or air after diving (DOCX 17 KB) [file 421_2022_4892_MOESM1_ESM.docx]

|  | | | **VGE max** | **KISS** |
| --- | --- | --- | --- | --- |
| **Treatment** | **Sample** | **Variable** | **Spearmans correlation p-value n** | **Spearmans correlation p-value n** |
| **Breathing oxygen after diving** | **30–45 minutes after diving** | **GFAP, absolute value** | -0.05 0.78 n=32 | -0.10 0.57 n=32 |
|  |  | **NFL, absolute value** | 0.09 0.63 n=32 | 0.14 0.43 n=32 |
|  |  | **tau, absolute value** | 0.25 0.17 n=32 | 0.21 0.25 n=32 |
|  |  | **Absolute change of GFAP from before dive to sample** | 0.19 0.29 n=32 | 0.15 0.40 n=32 |
|  |  | **Absolute change of NFL from before dive to sample** | 0.45 0.0095 n=32 | 0.43 0.013 n=32 |
|  |  | **Absolute change of tau from before dive to sample** | 0.24 0.18 n=32 | 0.22 0.22 n=32 |
| **Breathing oxygen after diving** | **120 minutes after diving** | **GFAP, absolute value** | -0.02 0.93 n=32 | -0.01 0.94 n=32 |
|  |  | **NFL, absolute value** | 0.02 0.91 n=32 | -0.01 0.97 n=32 |
|  |  | **tau, absolute value** | -0.08 0.68 n=32 | -0.08 0.65 n=32 |
|  |  | **Absolute change of GFAP from before dive to sample** | 0.13 0.49 n=32 | 0.16 0.38 n=32 |
|  |  | **Absolute change of NFL from before dive to sample** | 0.20 0.27 n=32 | 0.15 0.41 n=32 |
|  |  | **Absolute change of tau from before dive to sample** | -0.19 0.29 n=32 | -0.22 0.23 n=32 |
| **Breathing air after diving** | **30–45 minutes after diving** | **GFAP, absolute value** | 0.29 0.11 n=32 | 0.27 0.14 n=32 |
|  |  | **NFL, absolute value** | 0.32 0.073 n=32 | 0.33 0.064 n=32 |
|  |  | **tau, absolute value** | 0.12 0.52 n=32 | 0.07 0.70 n=32 |
|  |  | **Absolute change of GFAP from before dive to sample** | -0.21 0.24 n=32 | -0.20 0.28 n=32 |
|  |  | **Absolute change of NFL from before dive to sample** | -0.23 0.20 n=32 | -0.25 0.16 n=32 |
|  |  | **Absolute change of tau from before dive to sample** | 0.03 0.85 n=32 | 0.06 0.75 n=32 |
| **Breathing air after diving** | **120 minutes after diving** | **GFAP, absolute value** | 0.32 0.072 n=32 | 0.29 0.10 n=32 |
|  |  | **NFL, absolute value** | 0.35 0.052 n=32 | 0.35 0.048 n=32 |
|  |  | **tau, absolute value** | 0.15 0.41 n=32 | 0.16 0.38 n=32 |
|  |  | **Absolute change in GFAP concentration from before dive to sample** | -0.24 0.18 n=32 | -0.17 0.34 n=32 |
|  |  | **Absolute change of NFL from before dive to sample** | -0.15 0.42 n=32 | -0.21 0.25 n=32 |
|  |  | **Absolute change of tau from before dive to sample** | 0.31 0.088 n=32 | 0.38 0.030 n=32 |
| For each variable, the spearman's correlation is presented with corresponding p-value and number of observations | | | | |
