# Supplementary material for: Protein tau concentration in blood increases after SCUBA diving: an observational study
Source: Eur J Appl Physiol. 2022 Feb 10;122(4):993–1005. doi: 10.1007/s00421-022-04892-9 (PMC8926952; doi:10.1007/s00421-022-04892-9)
Supplement: Supplementary file 2 — Appendix 2: Spearmans correlation for GFAP, NfL, tau and their changes with VGE max and KISS30min or KISS120min after each dive (DOCX 17 KB) [file 421_2022_4892_MOESM2_ESM.docx]

| **Variable (pg·mL^-1^)** | **VGE_max_** | **KISS_30 min_** | |
| --- | --- | --- | --- |
| **GFAP (sample 2)** | 0.337 0.059 n=32 | 0.316 0.078 n=32 | |
| **GFAP (change from sample 1 to sample 2)** | 0.007 0.97 n=32 | -0.028 0.88 n=32 | |
| **NFL (sample 2)** | 0.417 0.017 n=32 | 0.433 0.013 n=32 | |
| **NFL (change from sample 1 to sample 2)** | -0.066 0.72 n=32 | -0.091 0.62 n=32 | |
| **Tau (sample 2)** | 0.050 0.79 n=32 | -0.044 0.81 n=32 | |
| **Tau (change from sample 1 to sample 2)** | 0.158 0.39 n=32 | 0.147 0.42 n=32 | |
| For each variable the spearman's correlation with corresponding p-value and number of observations are presented. | | | |
| **Variable (pg·mL^-1^)** | **VGE_max_** | **KISS_120min_** | |
| **GFAP (sample 3)** | 0.376 0.034 n=32 | 0.361 0.043 n=32 | |
| **GFAP (change from sample 1 to sample 3)** | -0.091 0.62 n=32 | -0.082 0.66 n=32 | |
| **NFL (sample 3)** | 0.456 0.0087 n=32 | 0.414 0.018 n=32 | |
| **NFL (change from sample 1 to sample 3)** | -0.020 0.91 n=32 | -0.103 0.57 n=32 | |
| **Tau (sample 3)** | 0.038 0.84 n=32 | 0.071 0.70 n=32 | |
| **Tau (change from sample 1 to sample 3)** | 0.277 0.12 n=32 | 0.321 0.073 n=32 | |
| For each variable the spearman's correlation with corresponding p-value and number of observations are presented. | | | |
| **Variable (pg·mL-1)** | **VGE_max_** | | **KISS_30 min_** |
| **GFAP (sample 5)** | -0.152 0.41 n=32 | -0.127 0.49 n=32 | |
| **GFAP (change from sample 4 to sample 5)** | 0.021 0.91 n=32 | 0.024 0.90 n=32 | |
| **NFL (sample 5)** | 0.040 0.83 n=32 | 0.090 0.62 n=32 | |
| **NFL (change from sample 4 to sample 5)** | 0.282 0.12 n=32 | 0.242 0.18 n=32 | |
| **Tau (sample 5)** | 0.232 0.20 n=32 | 0.223 0.22 n=32 | |
| **Tau (change from sample 4 to sample 5)** | 0.157 0.39 n=32 | 0.196 0.28 n=32 | |
| For each variable the spearman's correlation with corresponding p-value and number of observations are presented. | | | |
| **Variable (pg·mL^-1^)** | **VGE_max_** | **KISS_120min_** | |
| **GFAP (sample 6)** | -0.036 0.84 n=32 | -0.017 0.93 n=32 | |
| **GFAP (change from sample 4 to sample 6)** | 0.037 0.84 n=32 | 0.109 0.55 n=32 | |
| **NFL (sample 6)** | -0.067 0.72 n=32 | -0.031 0.87 n=32 | |
| **NFL (change from sample 4 to sample 6)** | 0.021 0.91 n=32 | -0.032 0.86 n=32 | |
| **Tau (sample 6)** | -0.007 0.97 n=32 | -0.036 0.84 n=32 | |
| **Tau (change from sample 4 to sample 6)** | -0.093 0.61 n=32 | -0.059 0.75 n=32 | |
| For each variable the spearman's correlation with corresponding p-value and number of observations are presented. | | | |
